# Interferon Gamma-Dependent Intestinal Pathology Contributes to the Lethality in Bacterial Superantigen-Induced Toxic Shock Syndrome

**DOI:** 10.1371/journal.pone.0016764

**Published:** 2011-02-03

**Authors:** Ashenafi Y. Tilahun, Marah Holz, Tsung-Teh Wu, Chella S. David, Govindarajan Rajagopalan

**Affiliations:** 1 Department of Immunology, Mayo Clinic College of Medicine, Rochester, Minnesota, United States of America; 2 Department of Anatomic Pathology, Mayo Clinic College of Medicine, Rochester, Minnesota, United States of America; McGill University, Canada

## Abstract

Toxic shock syndrome (TSS) caused by the superantigen exotoxins of *Staphylococcus aureus* and *Streptococcus pyogenes* is characterized by robust T cell activation, profound elevation in systemic levels of multiple cytokines, including interferon-γ (IFN-γ), followed by multiple organ dysfunction and often death. As IFN-γ possesses pro- as well as anti-inflammatory properties, we delineated its role in the pathogenesis of TSS. Antibody-mediated *in vivo* neutralization of IFN-γ or targeted disruption of *IFN-γ* gene conferred significant protection from lethal TSS in HLA-DR3 transgenic mice. Following systemic high dose SEB challenge, whereas the HLA-DR3.IFN-γ^+/+^ mice became sick and succumbed to TSS, HLA-DR3.IFN-γ^−/−^ mice appeared healthy and were significantly protected from SEB-induced lethality. SEB-induced systemic cytokine storm was significantly blunted in HLA-DR3.IFN-γ^−/−^ transgenic mice. Serum concentrations of several cytokines (IL-4, IL-10, IL-12p40 and IL-17) and chemokines (KC, rantes, eotaxin and MCP-1) were significantly lower in HLA-DR3.IFN-γ^−/−^ transgenic mice. However, SEB-induced T cell expansion in the spleens was unaffected and expansion of SEB-reactive TCR Vβ8^+^ CD4^+^ and CD8^+^ T cells was even more pronounced in HLA-DR3.IFN-γ^−/−^ transgenic mice when compared to HLA-DR3.IFN-γ^+/+^ mice. A systematic histopathological examination of several vital organs revealed that both HLA-DR3.IFN-γ^+/+^ and HLA-DR3.IFN-γ^−/−^ transgenic mice displayed comparable severe inflammatory changes in lungs, and liver during TSS. Remarkably, whereas the small intestines from HLA-DR3.IFN-γ^+/+^ transgenic mice displayed significant pathological changes during TSS, the architecture of small intestines in HLA-DR3.IFN-γ^−/−^ transgenic mice was preserved. In concordance with these histopathological changes, the gut permeability to macromolecules was dramatically increased in HLA-DR3.IFN-γ^+/+^ but not HLA-DR3.IFN-γ^−/−^ mice during TSS. Overall, IFN-γ seemed to play a lethal role in the immunopathogenesis of TSS by inflicting fatal small bowel pathology. Our study thus identifies the important role for IFN-γ in TSS.

## Introduction

Toxic shock syndrome (TSS) is a serious systemic illness caused by the Gram-positive cocci, *Staphylococcus aureus* or *Streptococcus pyogenes* [1], [2]. However, other organisms could be responsible for TSS as well [3]. Toxic shock syndrome caused by *S. aureus* and *S. pyogenes* could be either menstrual or non-menstrual, has a rapid onset and can result in mortality, if not treated promptly [4], [5], [6]. While *S. aureus* and *S. pyogenes* elaborate several exotoxins, the superantigen (SAg) exotoxins are directly implicated in the etiopathogenesis of TSS [7]. Mechanistically, the SAg produced by these bacteria bind directly to α or β chain of the MHC class II molecules, without undergoing any intracellular processing. Subsequently, the MHC class II-bound SAg robustly activate both CD4^+^ and CD8^+^ T cells by interacting directly with certain T cell receptor variable region β (TCR Vβ) chain families, irrespective of the antigen specificities of the T cells [8]. The T cells activated by SAg rapidly produce large amounts of cytokines and chemokines resulting in a sudden surge in the systemic levels of these biological mediators. This process, called systemic inflammatory response syndrome (SIRS), may lead to multiple organ dysfunction syndrome or MODS, wherein several vital organs within the body fail to perform their physiological functions. When MODS is not managed promptly, this will progress to irreversible end-stage organ failure and culminates in mortality. Apart from TSS, SAg also play an important role in the etiopathogenesis of several other acute systemic diseases caused by *S. aureus* and *S. pyogenes*, including sepsis [9], [10], [11]. In addition, certain superantigens, such as staphylococcal enterotoxin B (SEB), could be used as biological weapons [12]. Exposure to weaponized SEB can also causes a syndrome identical to TSS and can cause mortality.

The immunopathogenesis of TSS and related acute syndromes caused by SAg is poorly understood [13]. Currently, there are no specific therapies available to treat these clinical conditions and they are managed only by supportive therapy. Given that TSS is characterized by a robust elevation in systemic levels of several cytokines and chemokines, neutralization of key cytokines and/or chemokines could have therapeutic potential, as in other inflammatory diseases [14], [15]. In this context, while neutralization of TNF-α had been shown to be protective in animal models of TSS [16], clinical studies have shown that antagonizing the biological functions of TNF-α is ineffective during sepsis/TSS in human patients and might even be counter productive [17]. Therefore, other molecular targets need to be identified for treatment and/or prevention of TSS.

We and others have consistently observed that the expression of IFN-γ gene as well as systemic levels of IFN-γ are dramatically elevated during TSS [18], [19], [20], [21], [22], [23], [24], suggesting that IFN-γ could play an important role in the pathogenesis of TSS. However, the contradictory roles of IFN-γ in various inflammatory diseases have raised several unanswered questions regarding the pathogenic role for IFN-γ in TSS. IFN-γ has been traditionally considered as a pro-inflammatory cytokine responsible for eliciting immunopathology in several models of inflammation [25]. However, with the discovery of IL-17, and availability of newer reagents, a different role for IFN-γ has emerged in recent years. Unlike the previously held notions, IFN-γ has now been shown to protect from immunopathology in different models of inflammation (reviewed in Ref. [26]). For example, in the mouse model of multiple sclerosis, in vivo neutralization of IFN-γ resulted in exaggerated immune response and central nervous system immunopathology [27]. Similarly, in a mouse model of inflammatory bowel disease, IFN-γ has been shown to have anti-inflammatory role by suppressing IL-23 [28]. Collagen-induced arthritis is also exaggerated in the absence of IFN-γ signaling [29]. However, in the murine endotoxic shock model, IFN-γ seems to play a pathogenic role [30]. Similarly, IFN-γ has been shown to be lethal in lipopolysaccharide-sensitization models of TSS [31]. However, the role of IFN-γ in TSS without any additional sensitization protocols has not been thoroughly investigated using animal models that recapitulate human TSS. Also, the mechanisms by which IFN-γ either predisposes or protects from TSS have not been shown. Once the role of IFN-γ in the immunopathogenesis of TSS is clarified, novel clinical protocols could be developed either to treat or prevent TSS. With this background, we explored the role of IFN-γ in the immunopathogenesis of TSS using HLA-DR3 transgenic mice. Our study identifies a novel role for IFN-γ in the pathogenesis of TSS.

## Materials and Methods

### Mice

AE°.HLA-DR3 transgenic mice expressing the functional HLA-DRA1*0101 and HLA-DRB1*0301 transgenes on the complete mouse MHC class II-deficient background have been described earlier and were used in this study [18]. HLA-DR3 transgenic mice are extremely sensitive to TSS without the use of any sensitizing agents and faithfully recapitulate human TSS [19]. AE°.HLA-DR3.IFN-γ^−/−^ mice were generated by mating AE°.HLA-DR3 transgenic mice with C57BL6.IFN-γ^−/−^ mice (Jackson Laboratory, Bar Harbor, ME) as per standard breeding protocols. Mice expressing HLA-DR3 transgene and lacking endogenous MHC class II as well as IFN-γ were identified by PCR, selectively bred to generate the AE°.HLA-DR3.IFN-γ^−/−^ line. Mice were bred within the barrier facility of Mayo Clinic Immunogenetics Mouse Colony (Rochester, MN) and moved to a conventional facility after weaning. All the experiments were approved by the Mayo Clinic Institutional Animal Care and Use Committee (IACUC). The AAALAC Accreditation Number is 000717, the OLAW Assurance Number is A3291-01 and the study was covered under the protocols A29507 and A26408.

### Reagents

Endotoxin-reduced, highly purified staphylococcal enterotoxin B (SEB, Toxin Laboratories, Sarasota, FL) was dissolved in phosphate-buffered saline (PBS) at 1 mg/ml and stored frozen at -80°C in aliquots. Affinity purified goat anti murine-IFN-γ neutralizing antibodies (AF-485-NA) and goat IgG isotype control were purchased from R&D systems (Minneapolis, MN). The monoclonal rat anti-mouse IFN-γ antibody (clone HB-170) was purified from culture supernatants of hybridoma cell line grown in roller bottles as per standard procedure at Mayo antibody core facility (Mayo Clinic, Rochester, MN).

### SEB injections and response

For serum cytokine analyses, HLA-DR3.IFN-γ^+/+^ and HLA-DR3.IFN-γ^−/−^ transgenic mice were challenged with 10 µg of SEB in 200 µl PBS by intra-peritoneal route, bled at 3 hours. For in vivo IFN-γ neutralization, mice were first challenged 5 µg of SEB in 200 µl PBS. Immediately following this, mice received 100 µg of affinity purified goat anti-mouse IFN-γ neutralizing antibody (AF-485-NA) or 100 µg of goat IgG isotype control, all injected intra-peritoneally. Mice were bled at 3 hours for serum cytokine analysis using a multiplex bead assay, per the manufacturer's protocol using their software and hardware (Bio-Plex, Bio-Rad). For experiments involving flow cytometric analyses, mice were killed on specified days after SEB challenge and distribution of CD4^+^ and CD8^+^ T cells expressing TCR Vβ6 or TCR Vβ8 was determined in indicated tissues by flow cytometry using flurochrome conjugated antibodies (BD biosciences (San Jose, CA).

### Induction of toxic shock with SEB

Mice received either 50 or 100 µg of SEB in 200 µl PBS through intra-peritoneal route. Mice were closely monitored for the symptoms of shock. Rectal temperature was recorded at 0, 3, 6, 24, 48 and 72 hours. General physical activity of mice was scored on an arbitrary scale of 1–4, with 4 being normal activity and 1 being moribund (euthanized subsequently). For in vivo protection studies, mice were challenged with SEB (50 µg/mouse), immediately followed by indicated amounts of anti-IFN-γ monoclonal antibodies given by intra-peritoneal injection. Mice were closely monitored as above. Administration of D-galactosamine (D-galN) sensitizes mice to TSS and causes rapid death in a TNF-α-dependent manner [13], [16]. Therefore, we also investigated whether there is a differential susceptibility of HLA-DR3.IFN-γ^+/+^ and HLA-DR3.IFN-γ^−/−^ transgenic mice to D-galN-sensitized TSS. For this, mice were first injected with 5 µg of SEB in 200 µl PBS by intra-peritoneal injection, immediately followed by 30 mg of D-galN also injected through intra-peritoneal route.

### Gut permeability

Changes in intestinal permeability were determined using 4 KDa FITC-labeled dextran [32]. HLA-DR3.IFN-γ^+/+^ and HLA-DR3.IFN-γ^−/−^ mice were challenged with SEB (50 µg/mouse) as above. 24, 48 and 72 hours after SEB challenge, mice were deprived of food for 3 hours. Naïve mice were also deprived of food for 3 hours. All mice were then gavaged with FITC–labeled dextran (0.6 mg/G body weight). Subsequently, all mice were sacrificed 3 hours after gavage and blood was collected by cardiac puncture. Sera were separated by centrifugation and stored frozen at −70°C until time for analysis. Serum content of FITC-dextran was determined using a microplate reader to measure fluorescence (excitation, 490 nm; emission, 525 nm).

### Statistics

The statistical significance of the survival curves were determined by “Log-rank (Mantel-Cox) Test” using the software GraphPad Prism (version 3.0a; San Diego, CA). Other results were compared applying Student's t test using the same software.

## Results

### *In vivo* antibody-mediated neutralization of IFN-γ protects from SEB-induced TSS

**Figure 1:**
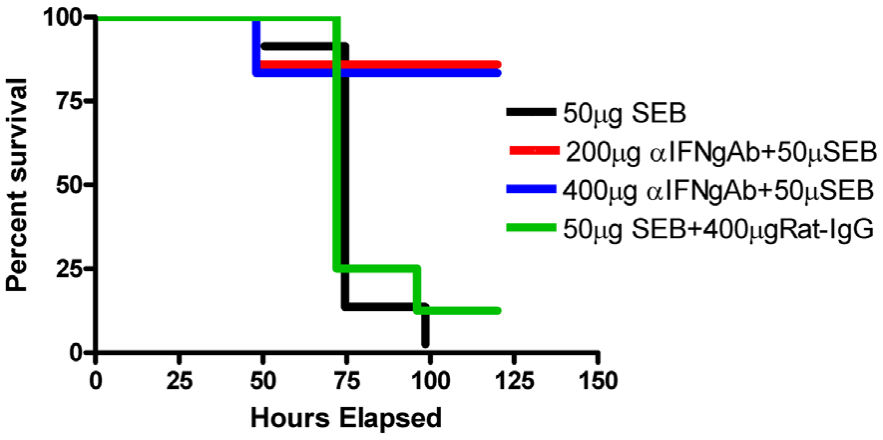
In vivo antibody mediated neutralization of IFN-γ protects from lethal SEB-induced TSS. Age-matched HLA-DR3 transgenic mice were challenged with SEB (50 µg) immediately followed by indicated amounts of monoclonal rat anti-mouse IFN-γ or isotype control. Mice were closely monitored for symptoms of TSS.

As shown in Fig. 1, HLA-DR3 transgenic mice challenged with SEB alone invariably succumbed to SEB-induced TSS as we have previously demonstrated. Similarly, HLA-DR3 transgenic mice challenged with SEB and treated with rat IgG isotype control antibodies also succumbed to TSS. On the contrary, only one out of six HLA-DR3 transgenic mice challenged with SEB and treated with anti-IFN-γ (400 µg/mouse) succumbed to TSS. Anti-IFN-γ mAb conferred similar protection from TSS even when the dose of antibody was reduced to 200 µg/mouse. This experiment clearly suggested that IFN-γ plays a lethal role in the immunopathogenesis of SAg-induced TSS and that antibody-mediated neutralization of IFN-γ could protect from TSS.

### Effect of antibody mediated neutralization of IFN-γ on SEB-induced cytokine and chemokine response *in vivo*

**Figure 2:**
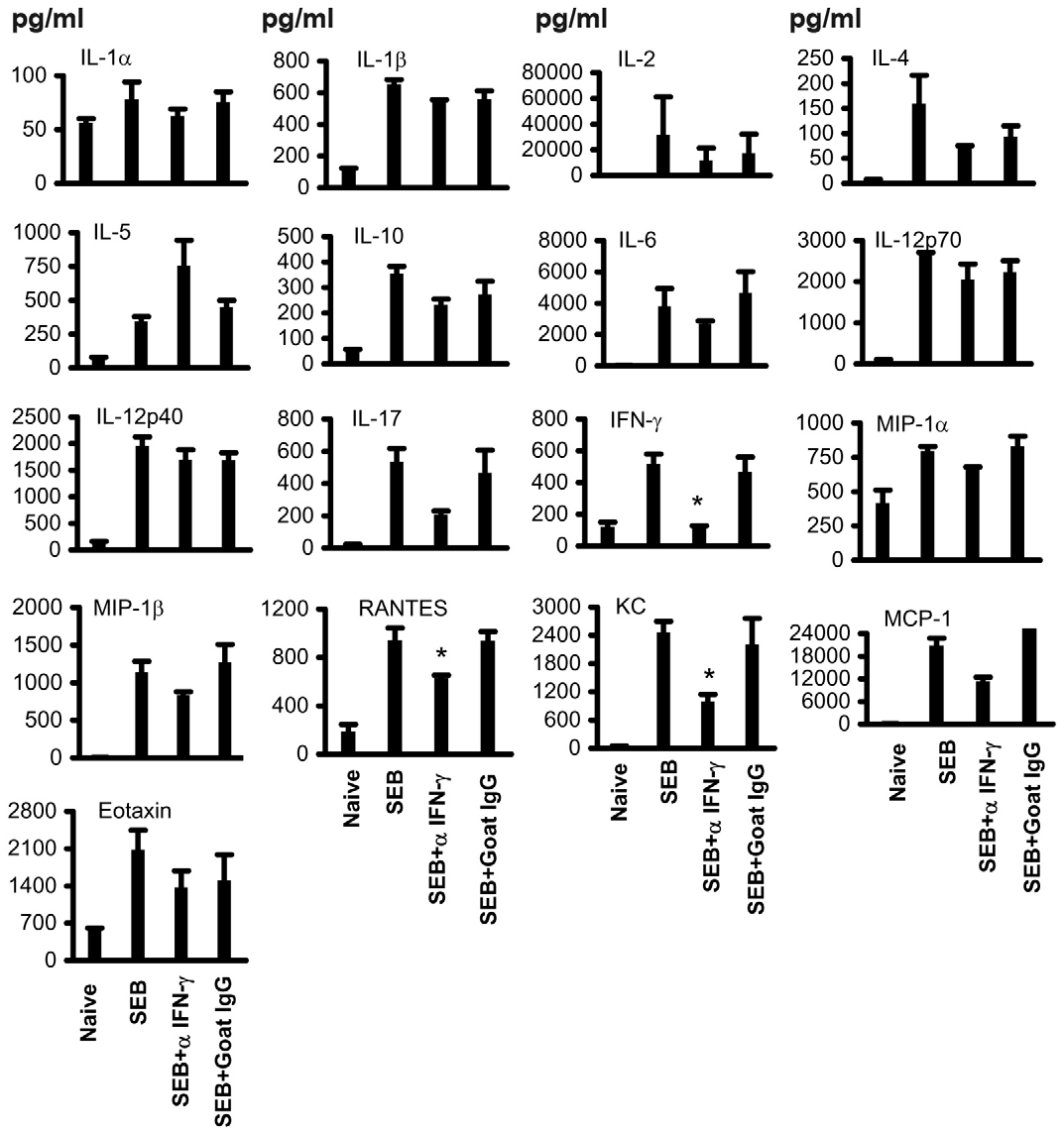
Effect of antibody mediated neutralization of IFN-γ on SEB-induced cytokine and chemokine responses in vivo. Age-matched HLA-DR3 transgenic mice were challenged with SEB (5 µg) immediately followed by 100 µg of goat anti-mouse IFN-γ or control goat antibodies. Mice were bled 3 hrs later and serum cytokine levels were determined using a multiplex suspension array system (Bio-Plex, Bio-Rad). Each bar represents the mean±SE of 4–6 mice.

As illustrated in Fig. 2, naïve HLA-DR3.IFN-γ^+/+^ mice had very low or undetectable levels of several cytokines/chemokines tested. Within 3 hours following SEB challenge, these cytokines/chemokines showed a significant elevation. Administration of neutralizing antibodies to IFN-γ immediately following SEB challenge caused changes in the concentrations of certain cytokines/chemokines in the serum. First of all, serum levels of IFN-γ were significantly reduced (p<0.05) when compared to SEB-challenged mice treated with isotype control. In addition, chemokines such as rantes (CCL5) and KC (CXCL1) were also significantly reduced. While SEB challenged mice treated with control IgG had nearly 450 to 500 pg/ml of IL-17 in their sera, anti-IFN-γ treated mice had a 2.2-fold reduction in the serum IL-17 levels (p  =  0.07). Similarly, serum levels of MCP-1 were markedly reduced in anti-IFN-γ treated mice when compared to isotype treated control mice, but this reduction was not statistically significant. Notably, anti-IFN-γ treated mice had nearly a 2-fold increase in serum IL-5 levels when compared to control IgG treated mice. Thus, in vivo neutralization of IFN-γ modulated the systemic levels of other cytokines/chemokines as well.

### Effect of antibody mediated neutralization of IFN-γ on SEB-induced splenic T cell expansion and thymocyte deletion

**Figure 3:**
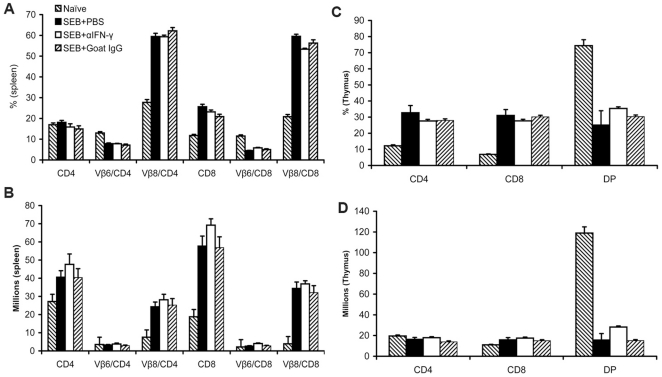
Effect of antibody mediated neutralization of IFN-γ on SEB-induced splenic T cell expansion and thymocyte deletion. Age-matched HLA-DR3 transgenic mice were challenged with SEB (5 µg) immediately followed by 100 µg of goat anti-mouse IFN-γ or control goat antibodies. Mice were killed 3 days later and distribution of different T cell subsets in the spleen (A and B) and thymus (C and D) was determined by flow cytometry. Each bar represents the mean±SE of 4–6 mice.

It is well established that following a single SEB challenge in vivo, both CD4^+^ and CD8^+^ mature T cells expressing TCR Vβ8 (SEB-reactive) but not Vβ6 (SEB non-reactive), expand in the spleens and lymph nodes by day 3. Therefore, we next investigated whether in vivo neutralization of IFN-γ had any effect on T cell expansion. As shown in Fig. 3A and B, when compared to naïve HLA-DR3 mice, mice that were challenged with SEB, as would be expected, had significantly increased numbers of both CD4^+^ and CD8^+^ mature T cells expressing TCR Vβ8 but not Vβ6. Spleens from mice challenged with SEB and treated anti-IFN-γ antibodies harbored more total CD4^+^ and CD8^+^ as well as respective TCR Vβ8^+^ T cells when compared to mice challenged with SEB alone or mice challenged with SEB and treated with isotype control antibodies. However, the differences were not statistically significant (Fig. 3B). Unlike the mature peripheral T cells, the immature CD4CD8 double positive thymocytes undergo massive deletion following a single injection of SEB by day 3 [33]. Thus, thymocyte deletion is also considered a hallmark of superantigen-mediated immune activation in vivo. As can be seen from Fig. 3C and D, while neutralization of IFN-γ appeared to confer some protection of CD4CD8 double positive T cells from apoptosis, this was not statistically significant. Overall, in vivo neutralization of IFN-γ had little inhibitory effect on SEB-induced expansion of mature T cells. This suggested that the protective effect of IFN-γ neutralization is not through inhibition of T cell activation or expansion.

### Targeted disruption of IFN-γ protects from SEB-induced TSS

**Figure 4:**
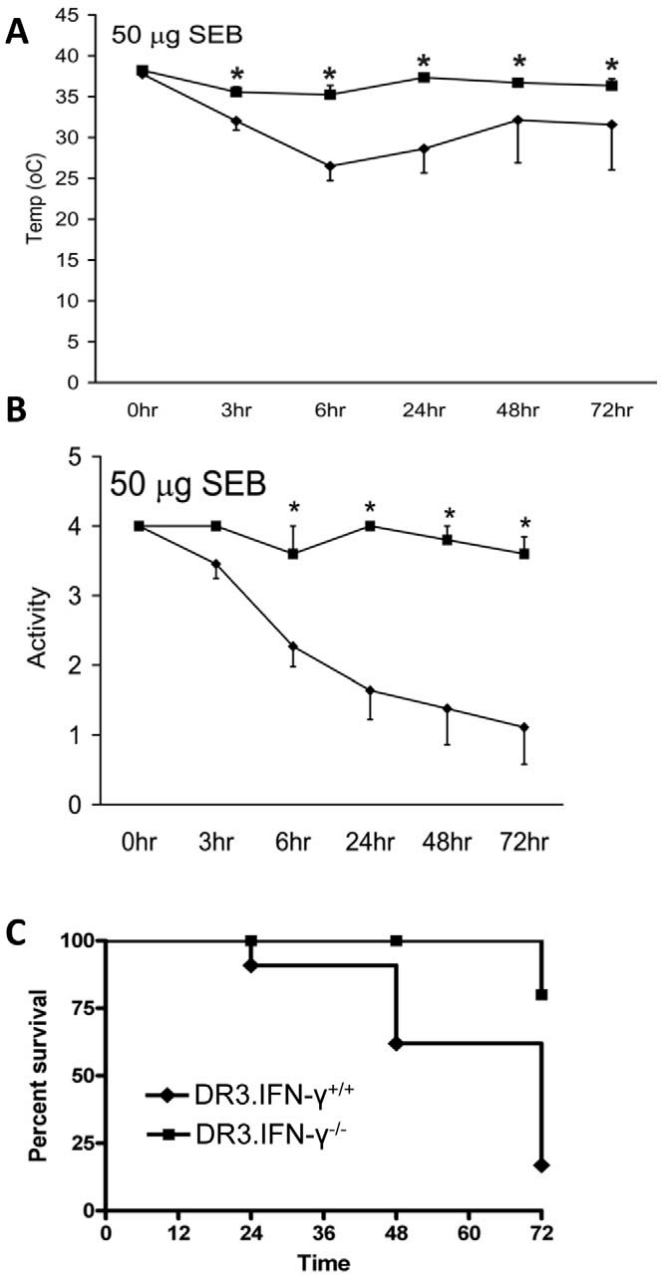
Effect of IFN-γ deficiency on SEB-induced TSS. Age-matched HLA-DR3.IFN-γ^+/+^ and HLA-DR3.IFN-γ^−/−^ transgenic mice were challenged with SEB (50 µg) and monitored closely. Data from 6–13 mice in each group.

We next wished to delineate the mechanisms by which IFN-γ contributed to the pathogenesis of TSS. Due to several advantages, we used HLA-DR3 transgenic mice with targeted disruption of *IFN-γ* gene for these studies. First of all, we confirmed the detrimental role for IFN-γ in SEB-induced TSS. HLA-DR3.IFN-γ^+/+^ and HLA-DR3.IFN-γ^−/−^ mice were challenged with 50 µg of SEB and monitored closely. As shown in Fig. 4, HLA-DR3.IFN-γ^+/+^ mice challenged with SEB became hypothermic, lethargic and 11 of the 13 mice died of TSS by 72 hours. On the other hand, the HLA-DR3.IFN-γ^−/−^ mice remained healthy, maintained their body temperature and maintained normal physical activity (p<0.05, Fig. 4A and B). Moreover, only 1 out of the 10 HLA-DR3.IFN-γ ^−/−^ mice died of TSS (p = 0.0007, Fig. 4C). HLA-DR3.IFN-γ^−/−^ resisted lethal SEB-induced TSS even when the dose of SEB was doubled to 100 µg/mouse. While the HLA-DR3.IFN-γ^+/+^ became progressively sick, the HLA-DR3.IFN-γ^−/−^ mice remained healthy throughout the study (Fig. S1). While 4 out of 4 HLA-DR3.IFN-γ^+/+^ transgenic mice died within the 3-day follow-up, only 1 out of 6 HLA-DR3.IFN-γ^−/−^ transgenic mouse died of TSS (p = 0.001). These results confirmed that IFN-γ plays a detrimental role in the in the pathogenesis of TSS.

### Interferon-γ deficiency modulates SEB-induced acute cytokine/chemokine production *in vivo*

**Figure 5:**
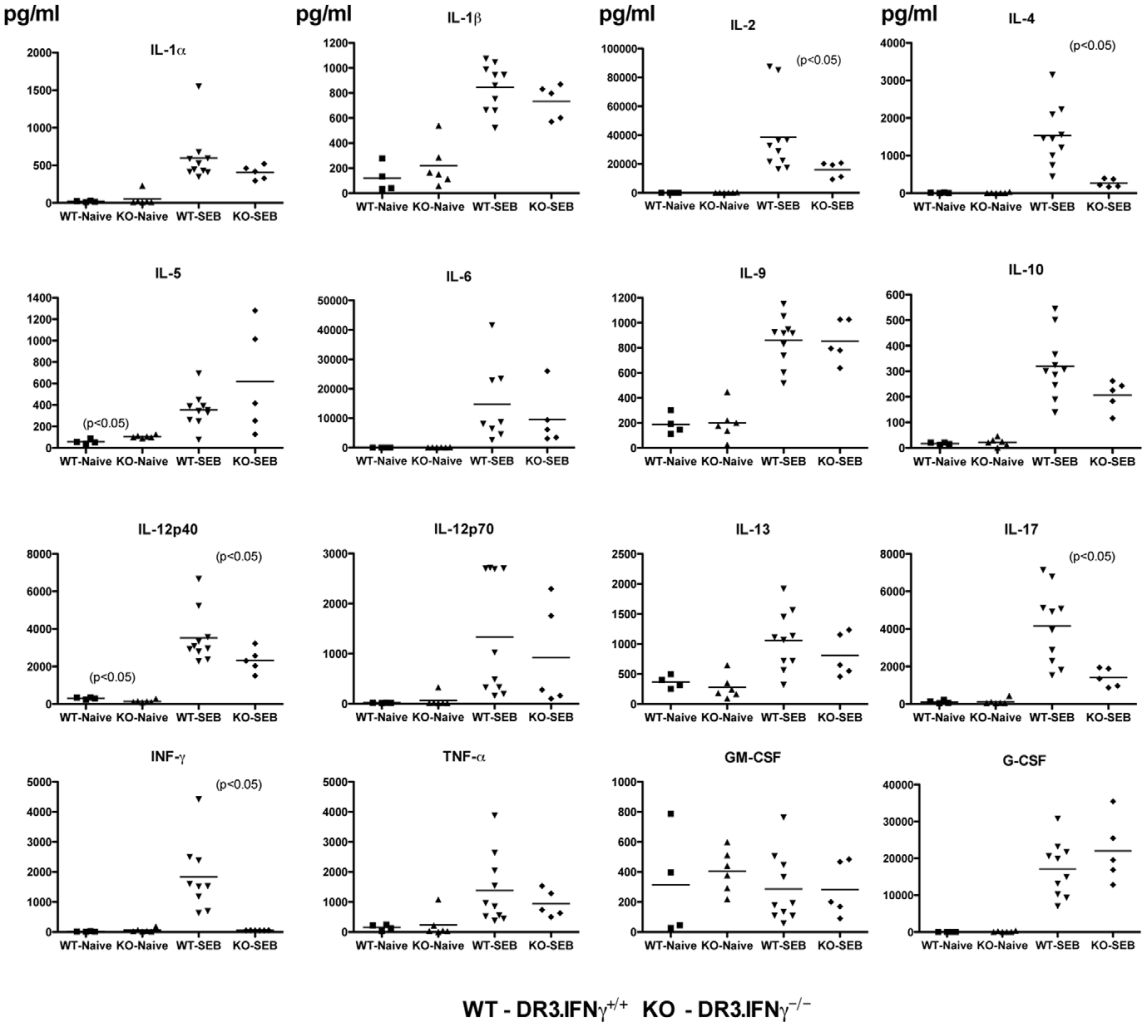
Effect of IFN-γ deficiency on SEB-induced cytokine responses in vivo. Age-matched HLA-DR3.IFN-γ^+/+^ and HLA-DR3.IFN-γ^−/−^ transgenic mice were challenged with SEB (10 µg). Mice were bled at 3 hrs and serum cytokine levels were determined using a multiplex suspension array system (Bio-Plex, Bio-Rad).

**Figure 6:**
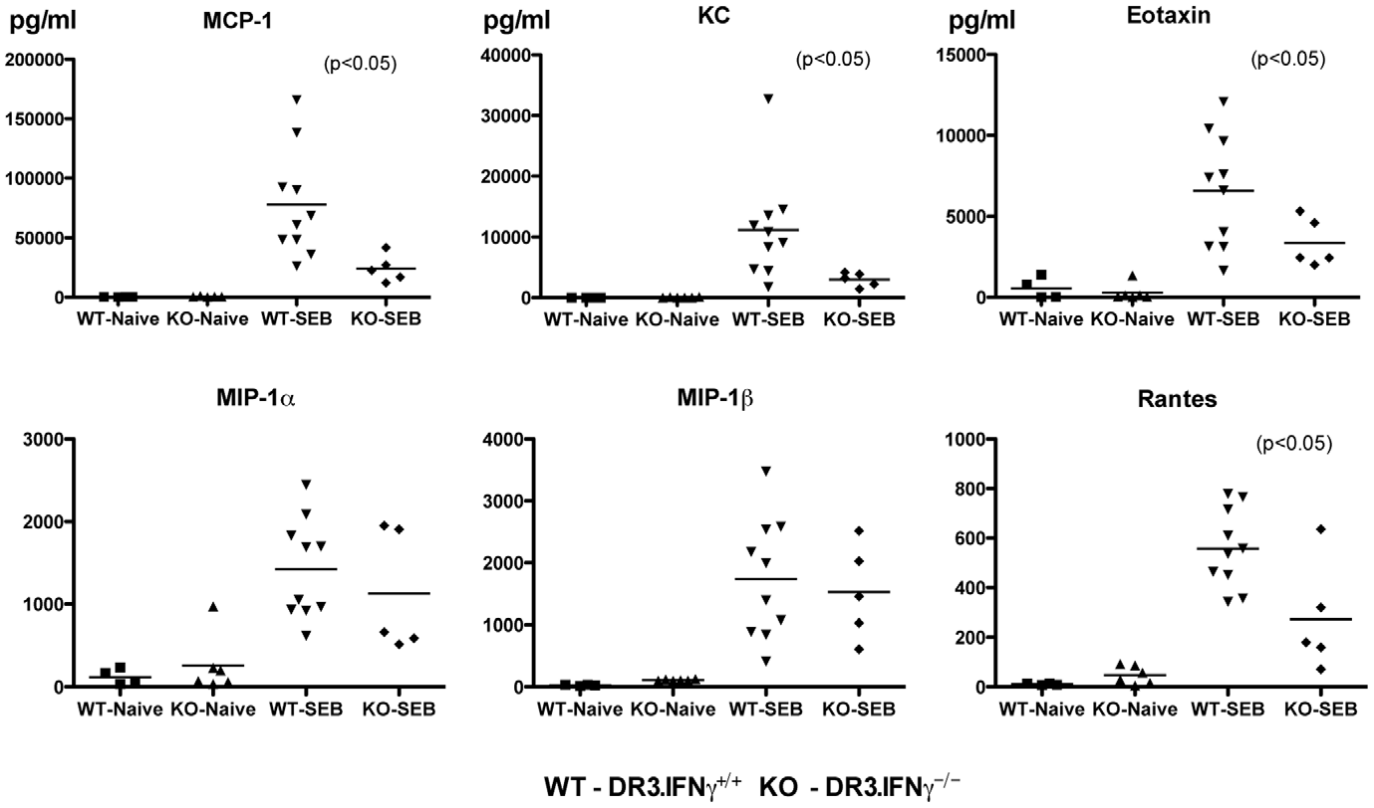
Effect of IFN-γ deficiency on SEB-induced chemokine responses in vivo. Age-matched HLA-DR3.IFN-γ^+/+^ and HLA-DR3.IFN-γ^−/−^ transgenic mice were challenged with SEB (10 µg). Mice were bled at 3 hrs and serum chemokine levels were determined using a multiplex suspension array system (Bio-Plex, Bio-Rad).

First of all, naïve HLA-DR3.IFN-γ^+/+^ mice had very low or undetectable levels of several cytokines/chemokines tested. Similarly, naïve HLA-DR3.IFN-γ^−/−^ mice also had undetectable levels of serum cytokines (Fig. 5) and chemokines (Fig. 6). Nonetheless, serum level of IL-5 was significantly higher in naïve HLA-DR3.IFN-γ^−/−^ when compared to their naïve HLA-DR3.IFN-γ^+/+^ counterpart (104.8±4.950 versus 58.00±10.49 pg/ml, respectively, P<0.05), while level of IL-12p40 was significantly lower in naïve HLA-DR3.IFN-γ^−/−^ when compared to their naïve HLA-DR3.IFN-γ^+/+^ counterpart (142.9±28.58 versus 310.2±28.39 pg/ml, respectively, P<0.05). We next explored if challenging with SEB brings about differential cytokine response in HLA-DR3.IFN-γ^+/+^ and HLA-DR3.IFN-γ^−/−^ transgenic mice.

Following SEB challenge, the serum levels of all the cytokines/chemokines were dramatically elevated in SEB-treated HLA-DR3.IFN-γ^+/+^ HLA-DR3 mice. Even in HLA-DR3.IFN-γ^−/−^ mice, serum levels of several cytokines/chemokines were elevated following SEB challenge. However, the fold inductions were different. Concentrations of certain cytokines were not significantly different between HLA-DR3.IFN-γ^+/+^ and HLA-DR3.IFN-γ^−/−^ mice, e.g., IL-1α, IL-1β, IL-3, IL-6, IL-9, G-CSF, TNF-α, MIP-1α and MIP-1β, (Fig. 5 and 6). Some cytokines/chemokines were significantly lower in SEB challenged HLA-DR3.IFN-γ^−/−^ mice. These included, IL-2 (p<0.04), IL-4 (p<0.002), IL-12p40 (p<0.05), IL-17 (p<0.005), KC (p<0.03) and Rantes (p<0.007). Some cytokines were higher in HLA-DR3.IFN-γ^−/−^, e.g., IL-5. The mean serum IL-5 concentration in SEB challenged HLA-DR3.IFN-γ^−/−^ mice was 2-fold higher when compared to HLA-DR3.IFN-γ^+/+^ mice but not significant (p = 0.07). As expected, serum IFN-γ concentration was undetectable (background level of the assay) in SEB challenged HLA-DR3.IFN-γ^−/−^ mice whereas it ranged from 700 to 4000 pg/ml (mean 1830±387 pg/ml) in SEB challenged HLA-DR3.IFN-γ^+/+^ mice. These results confirmed the known effects of IFN-γ that it can regulate the production of several cytokines and chemokines. More importantly, it should be noted that antibody mediated neutralization of IFN-γ also resulted in similar modulation in systemic cytokine/chemokines levels (Fig. 2). However, IFN-γ^−/−^ mice obviously expressed more profound changes as would be expected. It should be noted that TNF-α has been implicated as a key molecular player in the pathogenesis of TSS. However, we observed minimal changes in serum TNF-α levels between the TSS-susceptible HLA-DR3.IFN-γ^+/+^ and TSS-resistant HLA-DR3.IFN-γ^−/−^ mice.

### Interferon-γ deficient DR3 transgenic mice are susceptible to SEB-induced TSS with D-gal sensitization

SAg fail to bind to murine MHC class II molecules more effectively and hence mice are resistant to TSS. Therefore, conventional mice are artificially made susceptible to TSS by using sensitizing agents such as D-galN [13], [34]. It has been shown that TNF-α plays a very important role in the pathogenesis of D-galN sensitized TSS [16], [35]. Since HLA-DR3.IFN-γ^−/−^ transgenic mice still had elevated levels of TNF-α, we next studied whether these mice will be susceptible to TSS with D-galN sensitization. Interestingly, both HLA-DR3.IFN-γ^+/+^ (Mortality - 6/6) and HLA-DR3.IFN-γ^−/−^ (Mortality - 4/4) transgenic mice were equally susceptible to TSS with D-galN sensitization. Both HLA-DR3.IFN-γ^+/+^ and HLA-DR3.IFN-γ^−/−^ transgenic mice rapidly became hypothermic and failed to survive beyond 9 hours post-SEB challenge. The observations that HLA-DR3.IFN-γ^−/−^ transgenic mice are resistant to lethal TSS without D-galN sensitization which mimics human TSS, but are still susceptible TSS with D-galN sensitization underscored significant differences in the immunopathogenesis between the two models of TSS.

### Protection from SEB-induced TSS in HLA-DR3.IFN-γ^−/−^ transgenic mice is not due to defective T lymphocyte response to SEB

**Figure 7:**
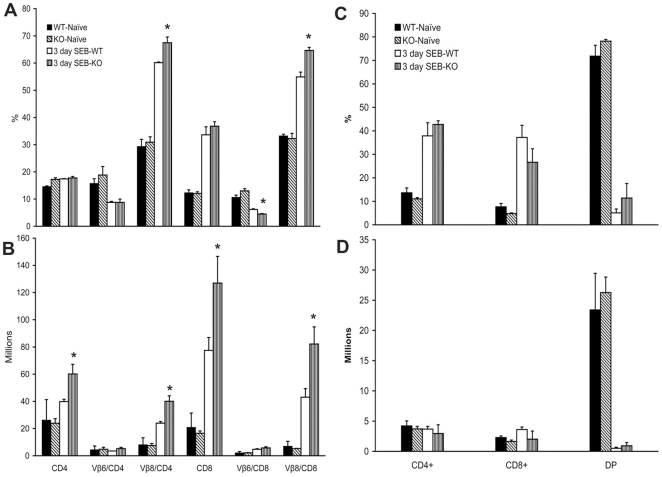
Effect of IFN-γ deficiency on SEB-induced splenic T cell expansion and thymocyte deletion. Age-matched HLA-DR3.IFN-γ^+/+^ and HLA-DR3.IFN-γ^−/−^ transgenic mice were challenged with SEB (10 µg). Mice were killed 3 days later and distribution of different T cell subsets in the spleen (A and B) and thymus (C and D) was determined by flow cytometry. Each bar represents the mean±SE of 4–6 mice.

IFN-γ deficiency did not limit the SEB-induced expansion of splenic TCR Vβ8^+^ T cells (Fig. 7A). Rather, there was a more robust expansion of TCR Vβ8^+^ but not Vβ6^+^ CD4 and CD8 T cells at day 3 in HLA-DR3.IFN-γ^−/−^ mice (Fig. 7A). When the percentages were converted to absolute numbers, the differences were more appreciable (Fig. 7B). With respect to the thymocytes (Fig. 7C and D), administration of SEB resulted in deletion of immature CD4CD8 double positive thymocytes equally in HLA-DR3.IFN-γ^+/+^ and HLA-DR3.IFN-γ^−/−^ transgenic mice. This indicated that protection from SEB-induced TSS is not due to defective T cell response to SEB in HLA-DR3.IFN-γ^−/−^ mice. On the other hand, IFN-γ appeared to dampen SEB-induced T cell expansion. In vitro stimulation of splenocytes from HLA-DR3.IFN-γ^+/+^ and HLA-DR3.IFN-γ^−/−^ mice showed similar responses in that HLA-DR3.IFN-γ^−/−^ splenocytes proliferated more vigorously to SEB (Fig. S2). Increased T cell proliferation in vitro or increased expansion of T cells in vivo in HLA-DR3.IFN-γ^−/−^ mice was probably not due to increased expression of HLA-DR3 molecules in HLA-DR3.IFN-γ^−/−^ mice because of comparable numbers of CD4^+^ T cells were present in both groups of mice in both thymus and spleen (Fig. 7). Similarly, the TCR Vβ8^+^ T cells were also not significantly different between naïve HLA-DR3.IFN-γ^+/+^ and HLA-DR3.IFN-γ^−/−^ transgenic mice (Fig. 7A and B).

In the next set of experiments, we studied if there are any defects in the kinetics of T cell activation in HLA-DR3.IFN-γ^−/−^ mice. For this, we collected splenocytes at 24, 48 and 72 hrs after SEB challenge from both HLA-DR3.IFN-γ^+/+^ and HLA-DR3.IFN-γ^−/−^ mice and studied the expression profile of activation markers such as CD69 and CD62L. Consistent with the above data, there appeared to be no defect in the kinetics of T cell activation in HLA-DR3.IFN-γ^−/−^ mice (Fig. S3). Overall, protection from TSS in HLA-DR3.IFN-γ^−/−^ mice was not due to defects in T cell activation.

### Effect of Interferon-γ deficiency on SEB-induced organ pathology

**Figure 8:**
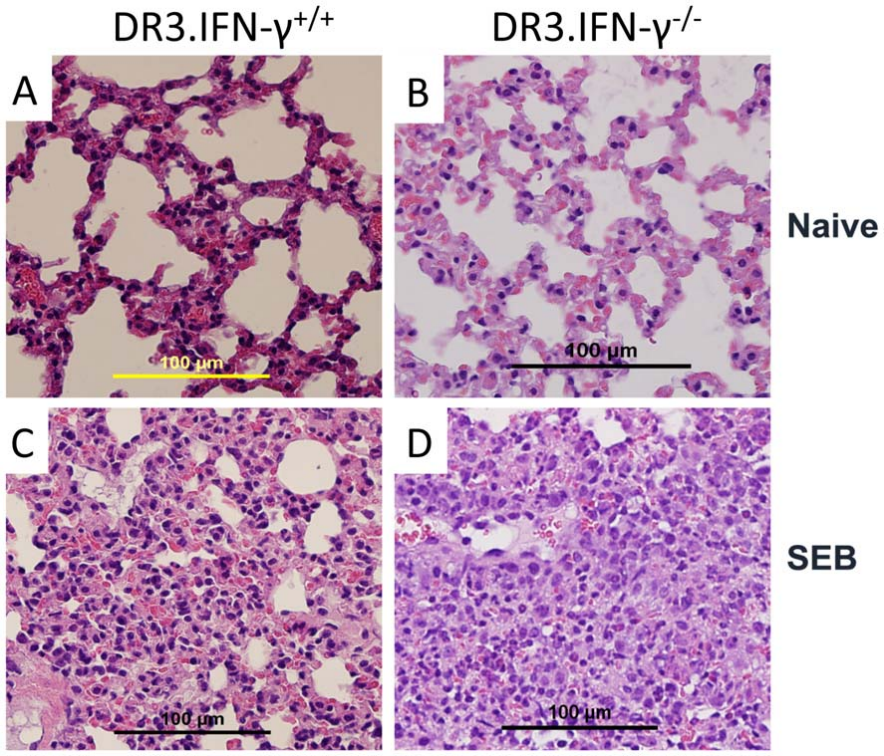
Effect of IFN-γ deficiency on SEB-induced pulmonary immunopathology. Age-matched HLA-DR3.IFN-γ^+/+^ (A and C) and HLA-DR3.IFN-γ^−/−^ (B and D) transgenic mice were left untreated (A and B) or challenged with SEB (50 µg, C and D). Mice were killed 48 hrs later, lungs were collected in buffered formalin and processed for H&E. The bar corresponds to 100 µM.

**Figure 9:**
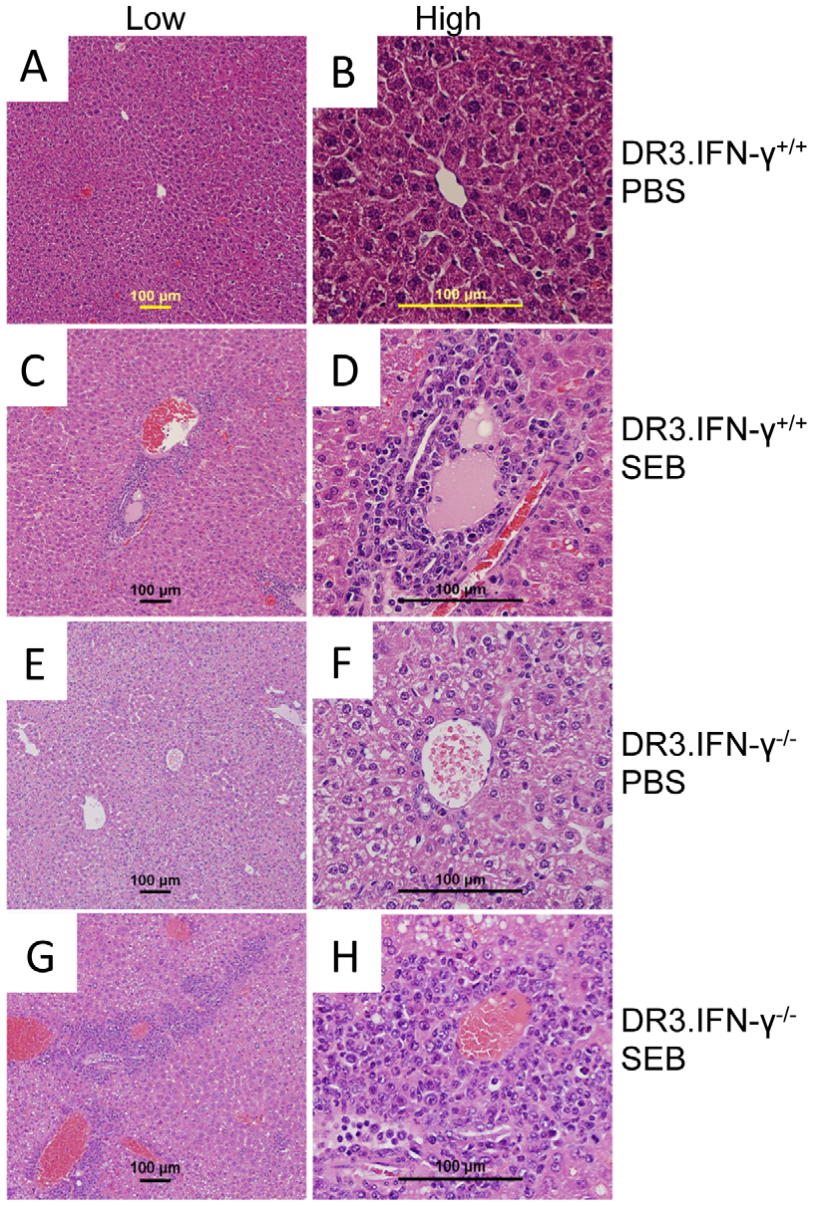
Effect of IFN-γ deficiency on SEB-induced hepatic immunopathology. Age-matched HLA-DR3.IFN-γ^+/+^ (A to D) and HLA-DR3.IFN-γ^−/−^ (E and H) transgenic mice were challenged with PBS (A, B and E, F) or challenged with SEB (50 µg) (C, D and G, H). Mice were killed 48 hrs later, livers were collected in buffered formalin and processed for H&E. The bar corresponds to 100 µM.

Toxic shock syndrome is characterized by multiple organ dysfunction. We have shown previously that HLA-DR3 transgenic mice challenged with SEB show significant multi-organ inflammation (involving lungs, liver, kidneys, heart and the intestines) analogous to human TSS. Since HLA-DR3.IFN-γ^−/−^ mice were protected from lethality associated with TSS, we next compared the extent of inflammatory changes in several organs between the HLA-DR3.IFN-γ^+/+^ and HLA-DR3.IFN-γ^−/−^ transgenic mice to gain some insights into the mechanisms by which IFN-γ plays a lethal role in TSS. As we have previously demonstrated, lungs (Fig. 8) and liver (Fig. 9) from HLA-DR3.IFN-γ^+/+^ mice challenged with SEB showed significant inflammatory changes. Surprisingly, these organs from HLA-DR3.IFN-γ^−/−^ mice challenged with SEB also showed significant inflammatory changes. These findings suggested that protection from SEB-induced lethality TSS in HLA-DR3.IFN-γ^−/−^ mice is not due to attenuated pathology in lungs and liver.

### Minimal intestinal pathology and conserved intestinal permeability in interferon-γ deficient mice during TSS

**Figure 10:**
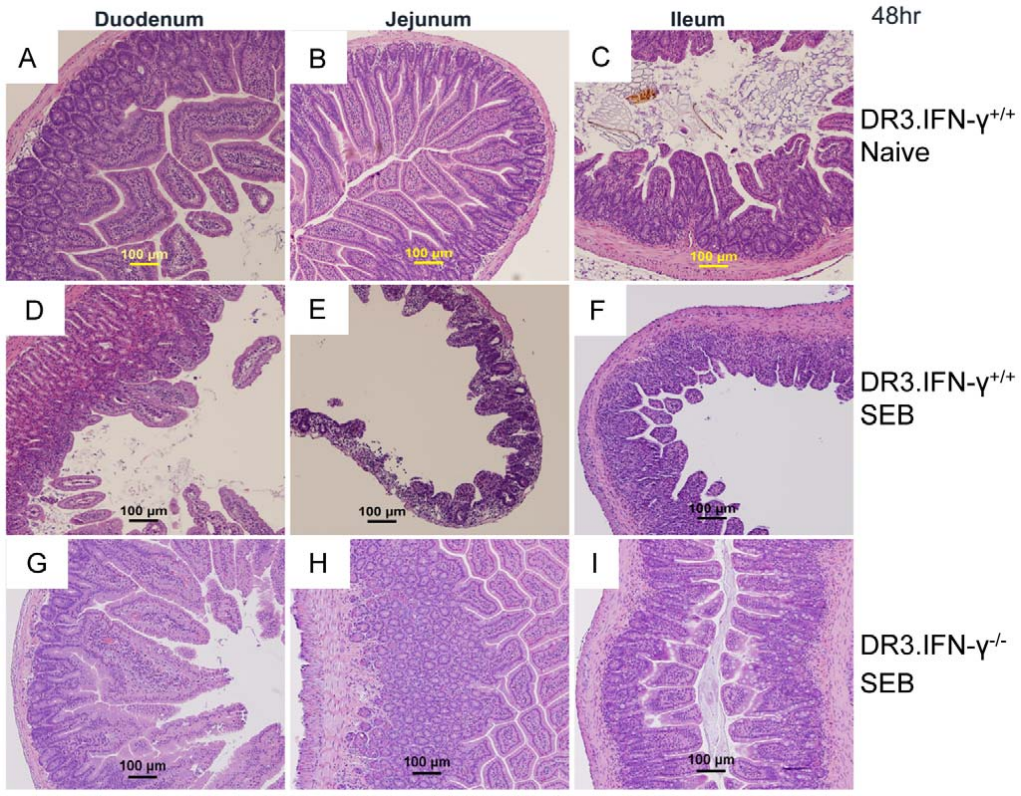
Effect of IFN-γ deficiency on SEB-induced intestinal immunopathology. Age-matched HLA-DR3.IFN-γ^+/+^ (D, E and F) and HLA-DR3.IFN-γ^−/−^ (G, H and I) transgenic mice were challenged with SEB (50 µg). Mice were killed 48 hrs later, intestinal segments were collected in buffered formalin and processed for H&E. Panels A, B and C – sections from naïve HLA-DR3.IFN-γ^+/+^ mice shown for comparison. The bar corresponds to 100 µM.

**Figure 11:**
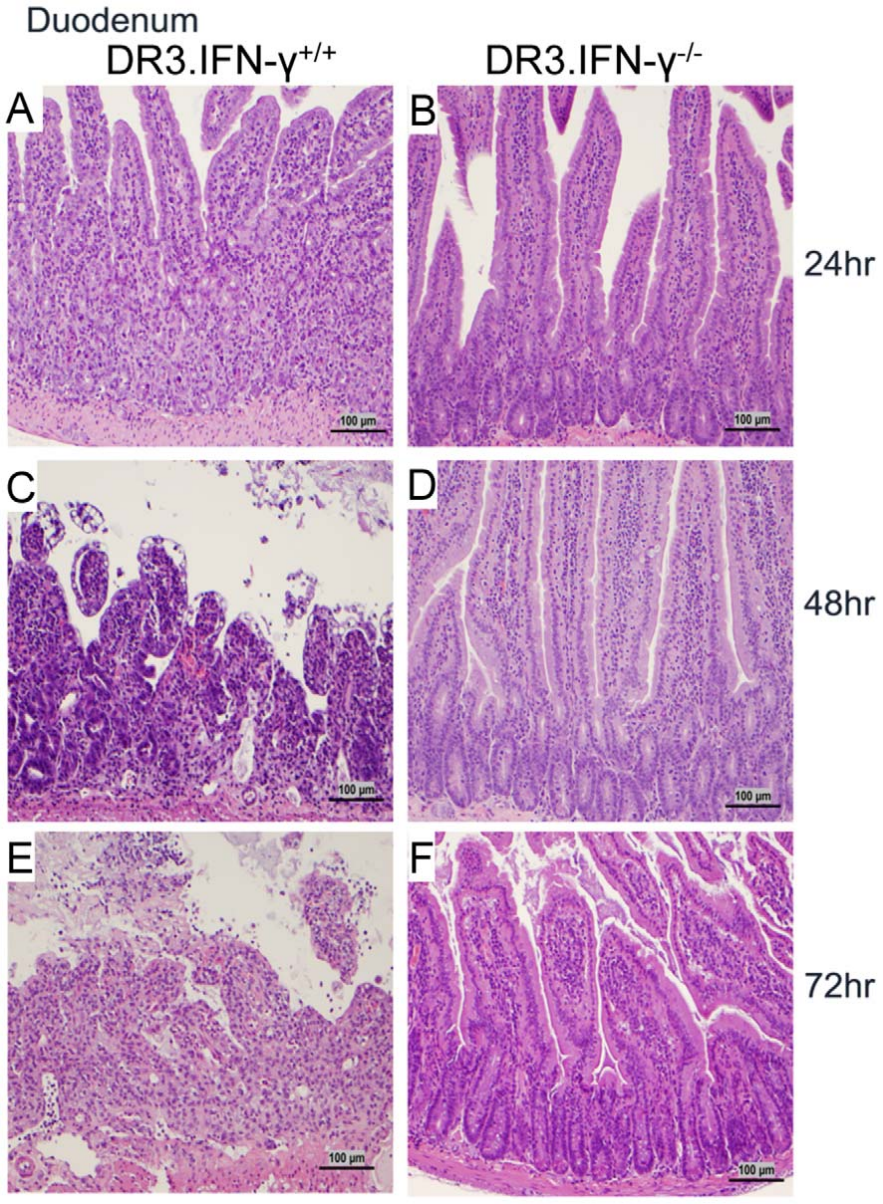
Effect of IFN-γ deficiency on SEB-induced duodenal immunopathology. Age-matched HLA-DR3.IFN-γ^+/+^ (A, C and E) and HLA-DR3.IFN-γ^−/−^ (B, D and F) transgenic mice were challenged with SEB (50 µg). Mice were killed at 24, 48 and 72 hrs, duodenal segments were collected in buffered formalin and processed for H&E. The bar corresponds to 100 µM.

One of the characteristic findings in SEB-induced TSS is extensive pathology to the small intestines, which is associated with changes in the gut permeability to macromolecules. Therefore, we next investigated whether reduced gut pathology could explain protection of HLA-DR3.IFN-γ^−/−^ transgenic mice from TSS. As previously demonstrated by us, HLA-DR3.IFN-γ^+/+^ transgenic mice challenged with SEB had significant pathological changes in the small intestine. There was dramatic shortening of villi in the small intestine. The integrity of the intestinal epithelial layer was completely altered. There was also complete loss of architecture of the lamina propria and submucosa throughout the small intestine. We could also detect the presence of large numbers of apoptotic cells in the intestinal epithelial layer. Large amounts of epithelial cell debris could be readily appreciated in the lumen of the small intestines from HLA-DR3.IFN-γ^+/+^ mice with TSS (Fig. 10, 11 and Figures S4 and S5). On the other hand, intestinal segments from HLA-DR3.IFN-γ^−/−^ mice challenged with SEB showed highly preserved histological architecture. There were minimal damages to the epithelial layer and the heights of the villi were preserved. Since the expression profiles of activation markers and expansion of T cells expressing TCR Vβ8 in HLA-DR3.IFN-γ^−/−^ was comparable to that seen in HLA-DR3.IFN-γ^+/+^ mice (Fig. S2), this rules out the possibility that subdued gut pathology in HLA-DR3.IFN-γ^−/−^ mice is due to defective T cell activation.

**Figure 12:**
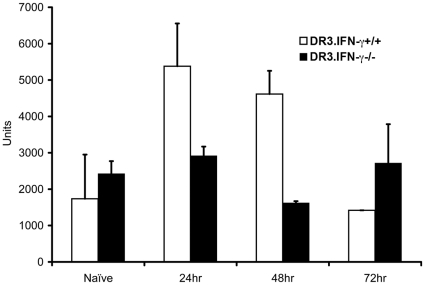
Effect of IFN-γ deficiency on SEB-induced alteration in gut permeability. Age-matched HLA-DR3.IFN-γ^+/+^ and HLA-DR3.IFN-γ^−/−^ transgenic mice were challenged with SEB (50 µg). Mice were gavaged with FITC-dextran as indicated in methods. Three hours after gavage, mice were killed, sera collected and the fluorescence in the sera were determined at 490 nm excitation and 525 nm emission.

In concordance with the histological changes in the gut, there was a significant change in gut permeability to macromolecules such as FITC-dextran in HLA-DR3.IFN-γ^+/+^ mice (Fig. 12). However, as expected, SEB challenged HLA-DR3.IFN-γ^−/−^ transgenic mice showed minimal changes in gut permeability as evidenced by lack of increase in fluorescence activity in sera at different time points. On the contrary, the fluorescence activity in the sera from HLA-DR3.IFN-γ^+/+^ transgenic mice significantly increased at 24 and 48 hours correlating with the gut pathology. However, at 72 hours, the fluorescent activity was reduced to baseline levels. This correlated with the loss of body weights in HLA-DR3.IFN-γ^+/+^ transgenic mice (Fig. 4). Combined with the histopathological picture, altered gut permeability and the significant drop in body weight in HLA-DR3.IFN-γ^+/+^ transgenic mice with time, it could be interpreted that there is a permanent damage to the gut epithelium as a result of which the absorptive functions of the epithelium is completely lost. Since HLA-DR3.IFN-γ^−/−^ mice did not show any significant pathological changes in the gut, maintained their body weights and preserved their gut permeability to macromolecules, we could conclude that IFN-γ-dependent gut pathology contributed to the lethality in TSS.

## Discussion

Toxic shock syndrome and other acute serious diseases that are particularly caused by *S. aureus* and *S. pyogenes* are attributed to the unique ability of their superantigen exotoxins to cause massive T cell activation and induce cytokine/chemokine secretion [36]. However, the exact molecular pathways by which these events lead to multiple organ dysfunction and ultimately death are not clearly understood [2]. As a result, there are no specific therapies available as of today to treat TSS. Since TSS is characterized by a systemic cytokine/chemokine storm, antagonizing or neutralizing the functions of selected pathogenic cytokines and/or chemokines could be beneficial. Given that numerous cytokines and chemokines are elevated during TSS, identifying a precise target with therapeutic utility could be a daunting task. Lack of appropriate animal models that closely recapitulate the human disease further complicates the problem.

It is well known that SAg bind more effectively with human MHC class II molecules than to mouse MHC class II molecules. Therefore, we have established that unlike the conventional mice strains, mice that transgenically express the HLA-DR or HLA-DQ molecules in the absence of any endogenous MHC class II molecules, mount a robust immune response to bacterial superantigens and are readily susceptible to TSS without the use of any sensitizing agents such as D-galactosamine or bacterial lipopolysaccharide (LPS) [18], [37], [38], [39], [40], [41]. TSS seen in HLA class II transgenic mice also closely mimics the human disease [22], [23]. Therefore, we used HLA-DR3 transgenic mice to evaluate the role of IFN-γ in the pathogenesis of TSS.

Antibody mediated neutralization of IFN-γ provided significant protection against lethal TSS indicating a critical role for IFN-γ in the lethality of TSS. Availability of HLA-DR3.IFN-γ^−/−^ mice facilitated the understanding of the mechanisms by which IFN-γ contributes to the lethality of TSS. Surprisingly, IFN-γ deficiency did not attenuate the extent of inflammation in lungs and liver. These observations concurred with the anti-inflammatory properties of IFN-γ [27], [28], [29]. Similarly, SAg-induced T cell expansion was more robust in HLA-DR3.IFN-γ^−/−^ mice. It is known that T cells from HLA-DR3.IFN-γ^−/−^ mice proliferate more robustly to antigen-specific and non-antigen-specific stimulation in vivo and in vitro [42], [43]. Therefore, it was not surprising to find increased T cell proliferation in HLA-DR3.IFN-γ^−/−^ mice. However, the difference in gut pathology between HLA-DR3.IFN-γ^+/+^ and HLA-DR3.IFN-γ^−/−^ mice provided some clues about possible pathways by which IFN-γ deficiency might have protected from lethality associated with TSS.

The involvement of gut in TSS has not been described earlier. Similarly, the role of IFN-γ in eliciting gut pathology in TSS has not been described before. However, IFN-γ is known to exert pathogenic effects on the intestinal epithelial cells, especially in the small intestine [44], [45]. For example, a recent study has shown that IFN-γ induces intestinal epithelial cell death. As a result, IFN-γ^−/−^ mice have conserved intestinal epithelial integrity following an inflammatory insult and therefore show increased survival [46], [47]. Similar observations were made in the current study. Paradoxically, IFN-γ deficiency did not affect inflammation in lungs or liver, but at the same time it protected small intestines from pathology. To our knowledge, such a contrasting effects of IFN-γ on different organs or systems has not been reported. It remains to be elucidated whether IFN-γ acts directly on the intestinal epithelial cells to bring forth the pathological changes or its effects are mediated indirectly through other molecular mediators. Unfortunately, the non-availability of KO mice with targeted disruption of IFN-γ receptor only on the intestinal epithelial cells precludes undertaking of any such studies. Nonetheless, several experiments done with isolated intestinal epithelial cells suggest a direct role for IFN-γ in eliciting intestinal epithelial cell pathology [48], [49]. D-galN sensitized TSS, has been shown to be a TNF-α mediated hepatotoxic process [50]. The susceptibility of HLA-DR3.IFN-γ^−/−^ mice to D-galN sensitized TSS further underscored the significance of IFN-γ in eliciting the small intestinal pathology. Taken together, our study suggests that IFN-γ-dependent small intestinal pathology and dysfunction, plays a significant role in the lethality of SAg-induced of TSS. The next step would be to verify if in vivo neutralization of IFN-γ is a viable therapeutic option in human TSS. Future studies using our mouse model would pave a strong foundation for such clinical studies regarding in vivo neutralization/antagonization of IFN-γ.

## Supporting Information

Figure S1 IFN-γ KO mice are protected from TSS induced by twice the lethal amount of SEB. Age-matched WT (HLA-DR3.IFN-γ^+/+^) and KO (HLA-DR3.IFN-γ^−/−^) DR3 mice were challenged with 100 µg of SEB. Body temperature, activity and mortality were determined as in Fig. 1.
(TIF)

Figure S2 Splenocytes from IFN-γ KO mice proliferate more vigorously to SEB stimulation in vitro. Splenocytes from age-matched WT (HLA-DR3.IFN-γ^+/+^) and KO (HLA-DR3.IFN-γ^−/−^) DR3 mice were cultured in vitro with SEB and cell proliferation was determined by thymidine incorporation assay. Representative data shown.
(TIF)

Figure S3 No defect in T cell activation in IFN-γ KO mice in response to SEB. Age-matched WT (HLA-DR3.IFN-γ^+/+^) and KO (HLA-DR3.IFN-γ^−/−^) DR3 mice were challenged with SEB. Splenocytes were analyzed by flowcytometry for expression profile of activation markers. Expression profile of CD69 shown.
(TIF)

Figure S4 Minimal intestinal immunopathology in KO DR3 mice. Age-matched WT (HLA-DR3.IFN-γ^+/+^) and KO (HLA-DR3.IFN-γ^−/−^) DR3 mice were challenged with a lethal dose of SEB. Intestinal segments were collected at indicated time points and evaluated by H&E staining.
(TIF)

Figure S5 Minimal intestinal immunopathology in KO DR3 mice. Age-matched WT (HLA-DR3.IFN-γ^+/+^) and KO (HLA-DR3.IFN-γ^−/−^) DR3 mice were challenged with a lethal dose of SEB. Figure shows representative images acquired at higher magnification from jejunal sections at 72 hrs.
(TIF)
