# Supplementary figures and images for: Interferon Gamma-Dependent Intestinal Pathology Contributes to the Lethality in Bacterial Superantigen-Induced Toxic Shock Syndrome
Source: PLoS One. 2011 Feb 3;6(2):e16764. doi: 10.1371/journal.pone.0016764 (PMC3033413; doi:10.1371/journal.pone.0016764)

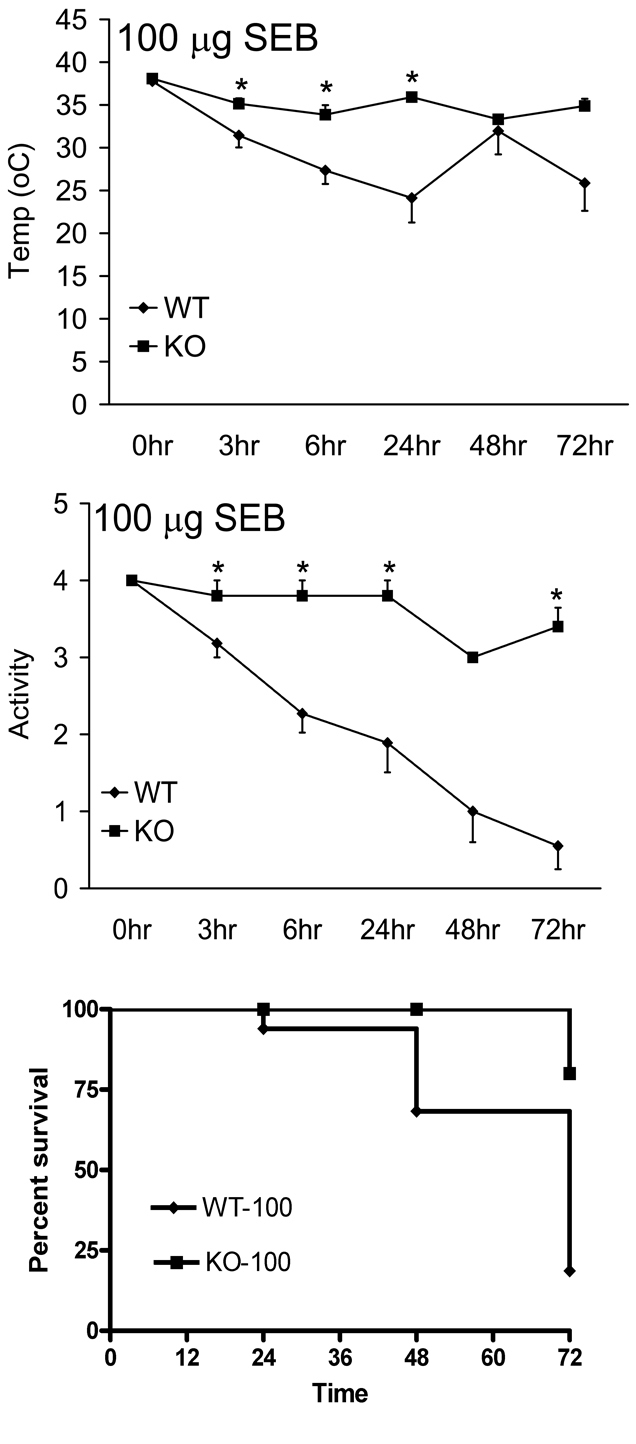

Supplement: Figure S1 — IFN-γ KO mice are protected from TSS induced by twice the lethal amount of SEB. Age-matched WT (HLA-DR3.IFN-γ+/+) and KO (HLA-DR3.IFN-γ−/−) DR3 mice were challenged with 100 µg of SEB. Body temperature, activity and mortality were determined as in Fig. 1. (TIF) [file pone.0016764.s001.tif]

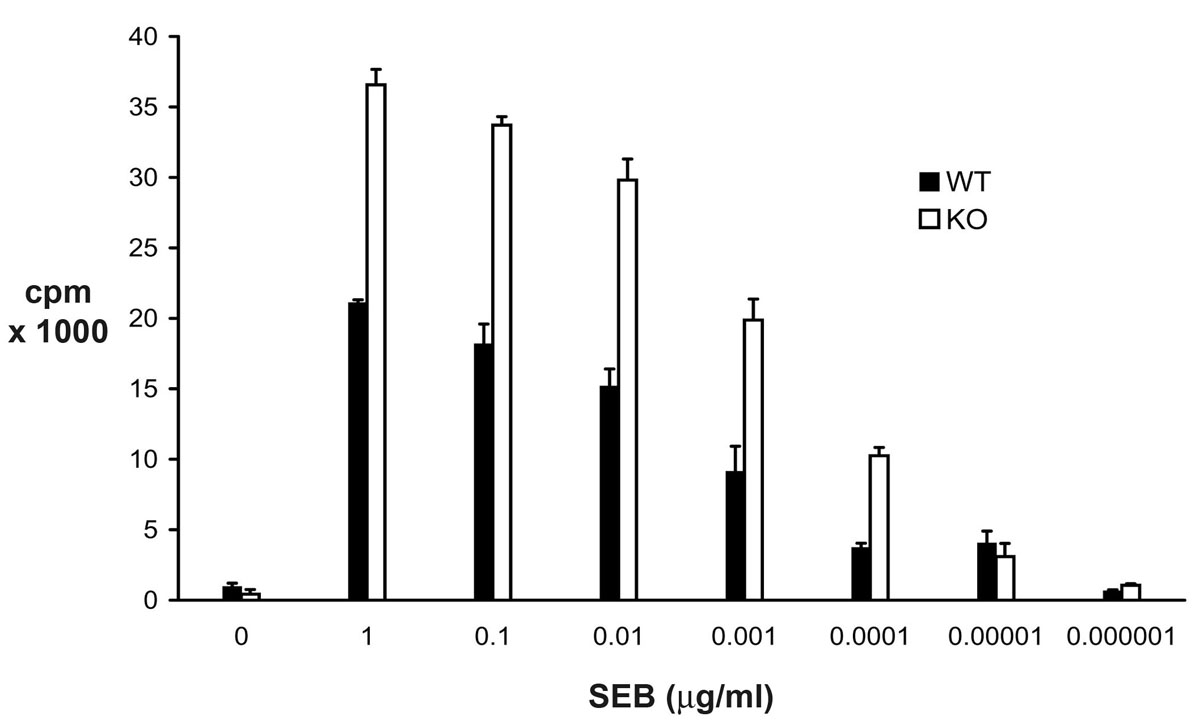

Supplement: Figure S2 — Splenocytes from IFN-γ KO mice proliferate more vigorously to SEB stimulation in vitro. Splenocytes from age-matched WT (HLA-DR3.IFN-γ+/+) and KO (HLA-DR3.IFN-γ−/−) DR3 mice were cultured in vitro with SEB and cell proliferation was determined by thymidine incorporation assay. Representative data shown. (TIF) [file pone.0016764.s002.tif]

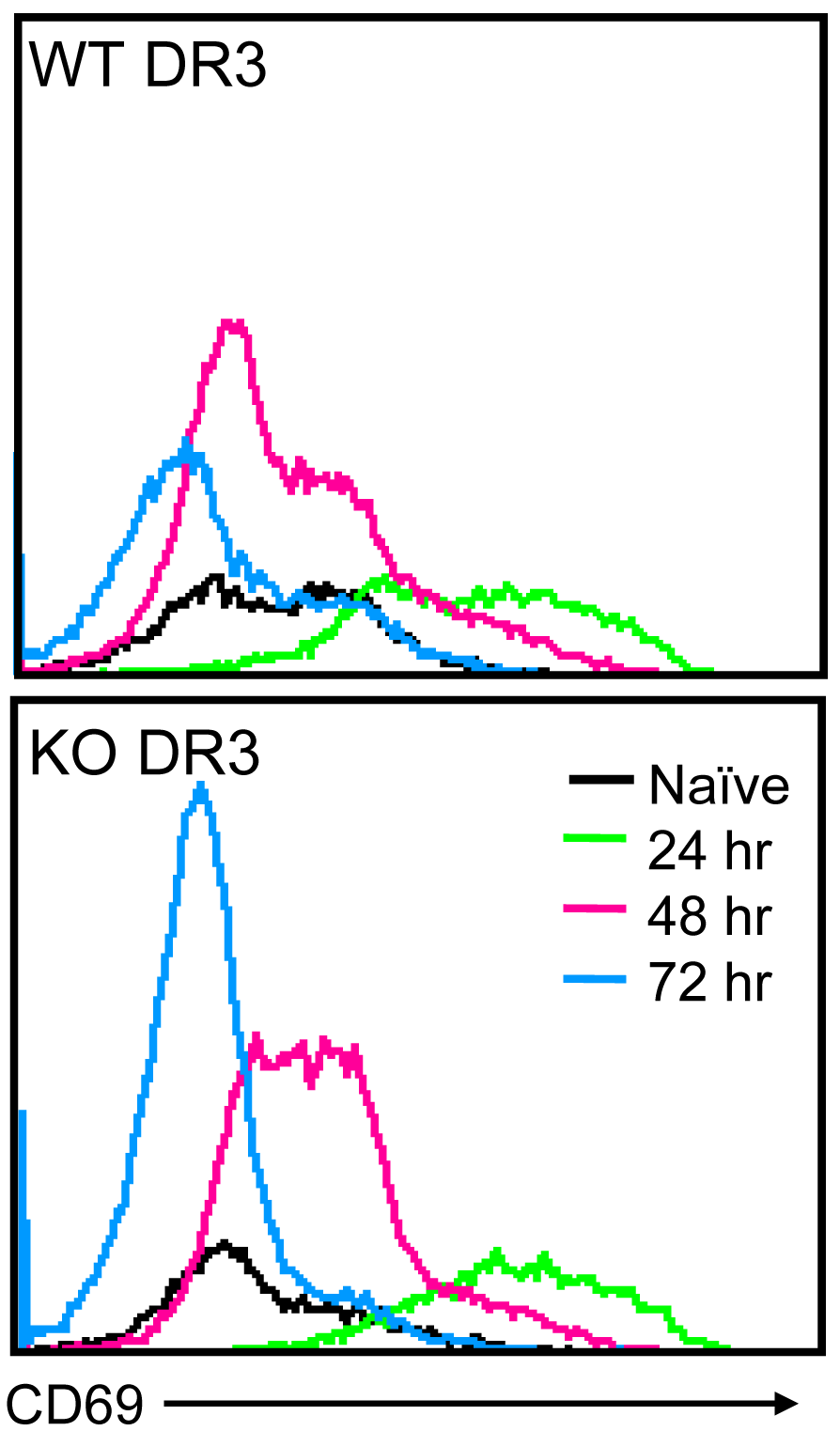

Supplement: Figure S3 — No defect in T cell activation in IFN-γ KO mice in response to SEB. Age-matched WT (HLA-DR3.IFN-γ+/+) and KO (HLA-DR3.IFN-γ−/−) DR3 mice were challenged with SEB. Splenocytes were analyzed by flowcytometry for expression profile of activation markers. Expression profile of CD69 shown. (TIF) [file pone.0016764.s003.tif]

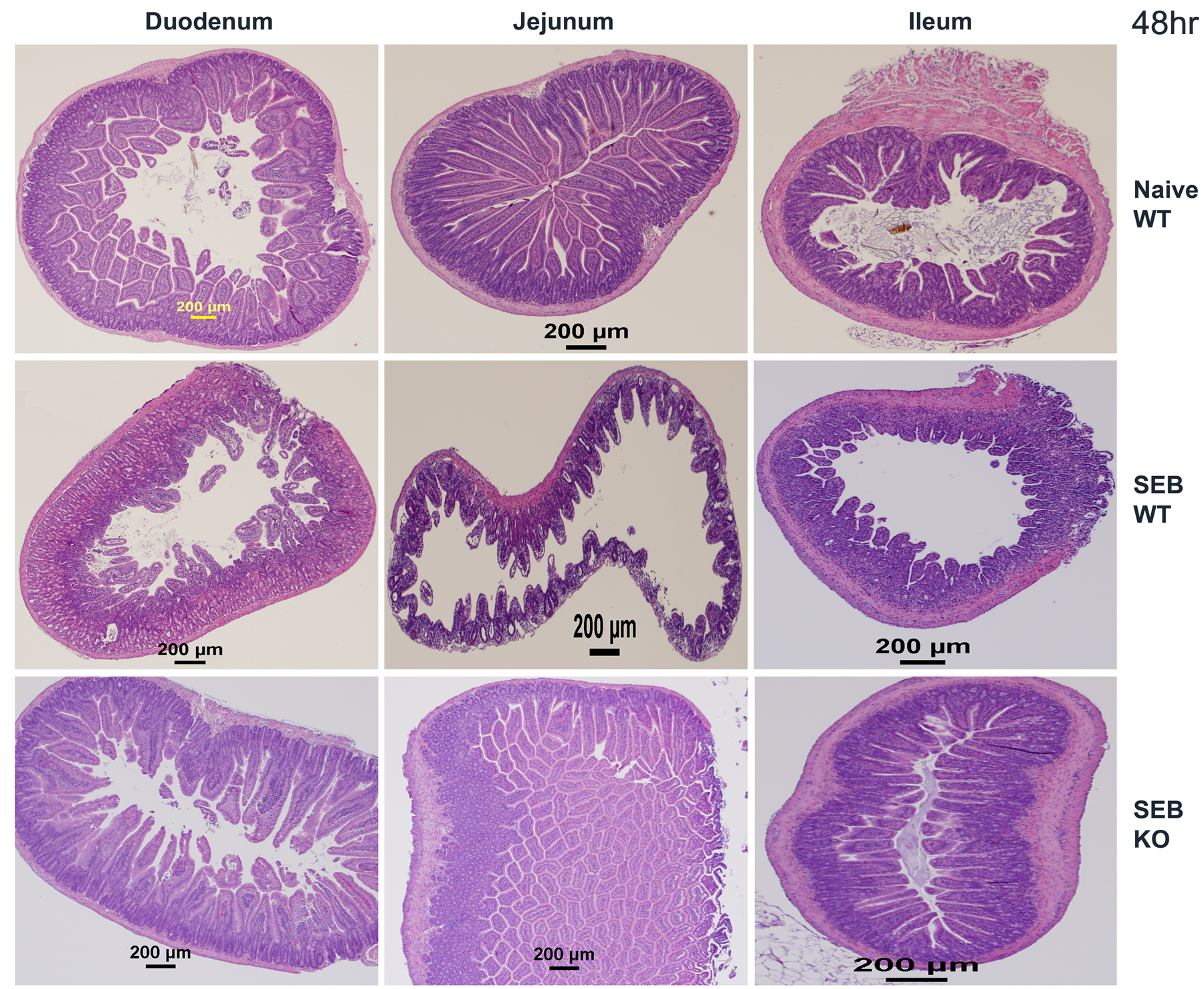

Supplement: Figure S4 — Minimal intestinal immunopathology in KO DR3 mice. Age-matched WT (HLA-DR3.IFN-γ+/+) and KO (HLA-DR3.IFN-γ−/−) DR3 mice were challenged with a lethal dose of SEB. Intestinal segments were collected at indicated time points and evaluated by H&E staining. (TIF) [file pone.0016764.s004.tif]

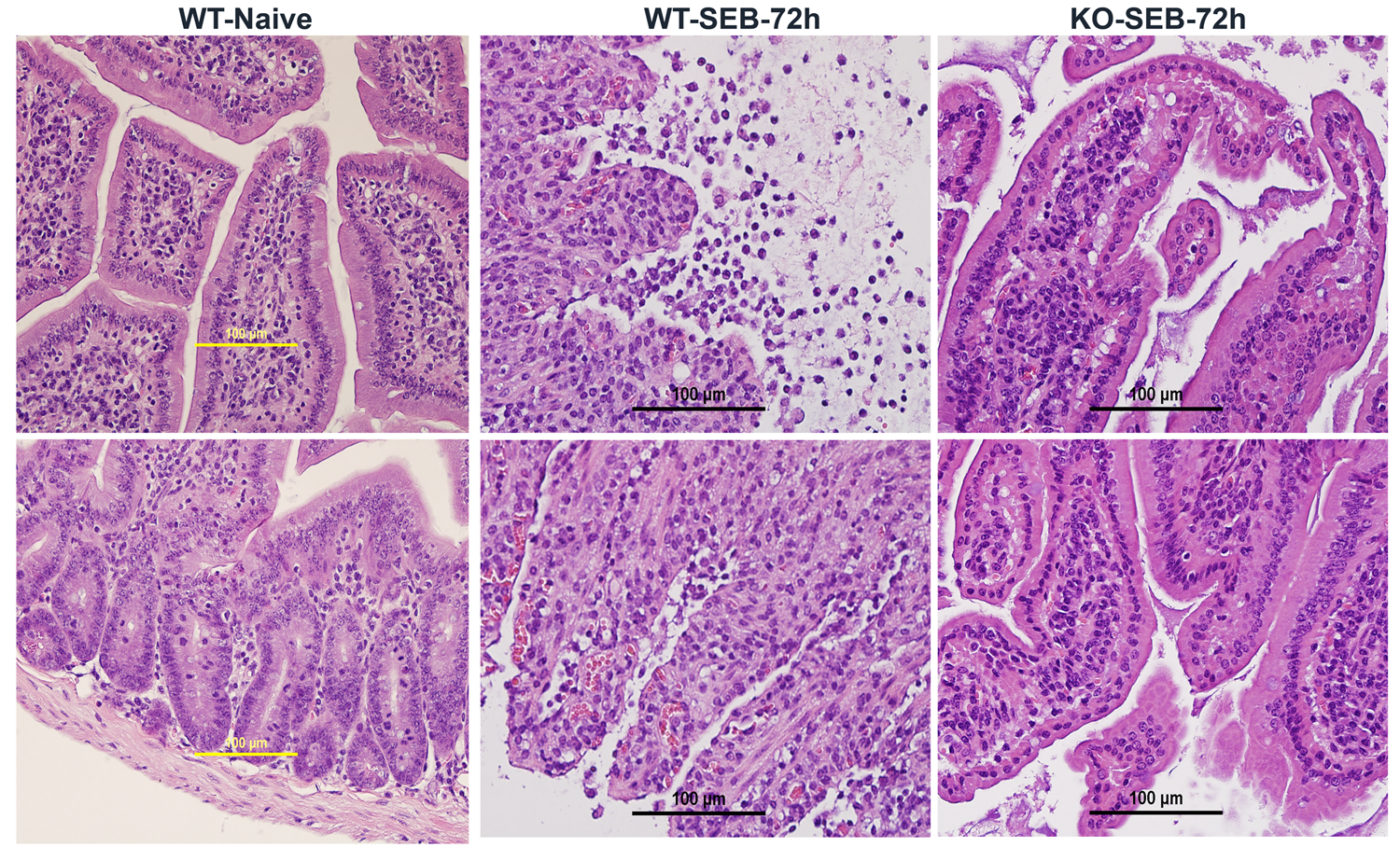

Supplement: Figure S5 — Minimal intestinal immunopathology in KO DR3 mice. Age-matched WT (HLA-DR3.IFN-γ+/+) and KO (HLA-DR3.IFN-γ−/−) DR3 mice were challenged with a lethal dose of SEB. Figure shows representative images acquired at higher magnification from jejunal sections at 72 hrs. (TIF) [file pone.0016764.s005.tif]
